# PB.28: Is second-time vacuum-assisted biopsy effective in improving preoperative diagnosis rate of screen-detected DCIS?

**DOI:** 10.1186/bcr3528

**Published:** 2013-11-08

**Authors:** S Pattison, A Kumar, W Teh, R Patel

**Affiliations:** 1 NLBSS, Edgware, UK

## Introduction

The NHSBSP standards for non-operative diagnosis are a target of 95% for invasive cancers (minimum 90%) and 90% for non-invasive cancer (minimum 85%). The lower target for non-invasive cancers reflects the difficulty in achieving definitive diagnosis of DCIS. Our unit has routinely used vacuum biopsies (VAB) for non-operative diagnosis of microcalcifications. Following an audit in 2009, repeat VAB were extended to B3 cases selected in MDT in order to improve non-operative diagnosis of DCIS.

## Methods

KC 62 report was used to identify all screen-detected cancers in 2011 and 2012. The mammographic characteristics, type and number of biopsy procedures and results were reviewed and correlated against final surgical outcomes. A final outcome of LCIS was excluded from the audit. The results were compared against a similar audit in 2008 and 2009.

## Results

In total, 86/330 (26.1%) were DCIS. A total of 90.7% (78/86) of non-invasive cancers were diagnosed preoperatively compared with 86% in 2008 and 2009. Non-operative diagnosis was achieved in 81.4% (70/86) on the first biopsy and 9.3% (8/86) on the second biopsy. Four cases of DCIS were completely excised by VAB. Six cases upgraded by repeat VAB were microcalcifications. Seven of eight cases of DCIS with no preoperative diagnosis were not subjected to second-time VAB.

## Conclusion

Second-time VAB successfully improve non-operative diagnosis of DCIS even when VAB are routinely used in the assessment of microcalcifications. The practice should be considered for all B3 cases in order to further improve the non-operative diagnosis rate for DCIS.

